# Supplementary material for: Cuticular profiling of insecticide resistant Aedes aegypti
Source: Sci Rep. 2023 Jun 22;13:10154. doi: 10.1038/s41598-023-36926-3 (PMC10287657; doi:10.1038/s41598-023-36926-3)
Supplement: Supplementary file 1 — Supplementary Figures. [file 41598_2023_36926_MOESM1_ESM.pdf]

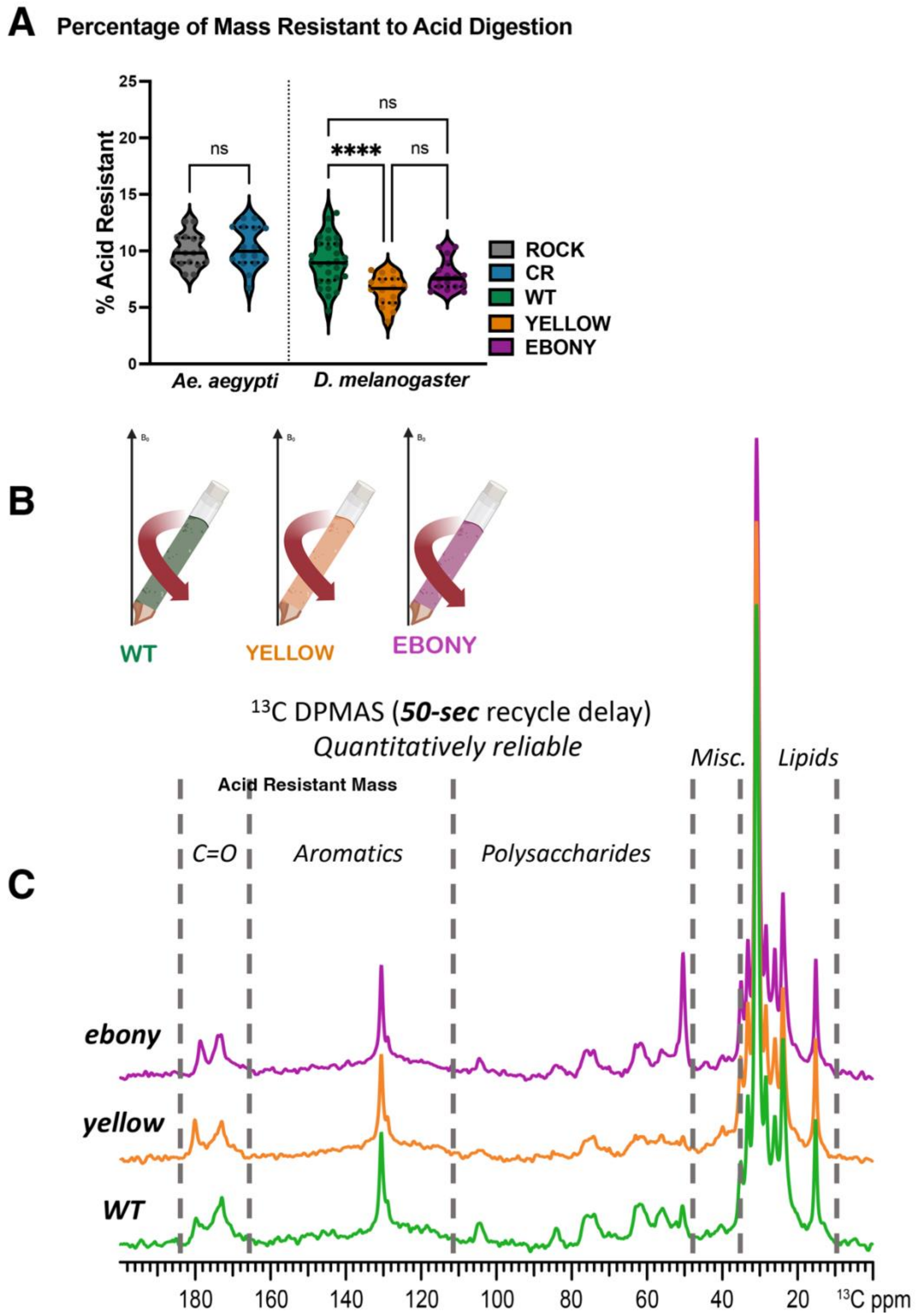

Supplementary Figure 1:

A: Percentage of female *Ae. aegypti* ROCK (grey) and CR (dark blue) and *D. melanogaster* WT (green), yellow (orange), and ebony (pink) wet weights that were resistant to acid digestion. All digestion samples contained 25 females each across three pooled biological replicates. Sample number: CR n = 16, ROCK n = 17, WT n= 28, yellow n= 16, Ebony n = 16. One-way ANOVA with

Tukey's Multiple Comparison test p value: ****= <0.0001, ** = 0.0048 **B:** Schematic of material loaded into ssNMR rotor to compare acid-resistant material from *D. melanogaster* strains **C:** direct-polarization (DPMAS) Carbon-13 (^{13}C) ssNMR (50-sec delay; quantitatively reliable) comparison of acid-resistant material of the WT (green), yellow (orange), and ebony (pink) strains pooled from three biological replicates.

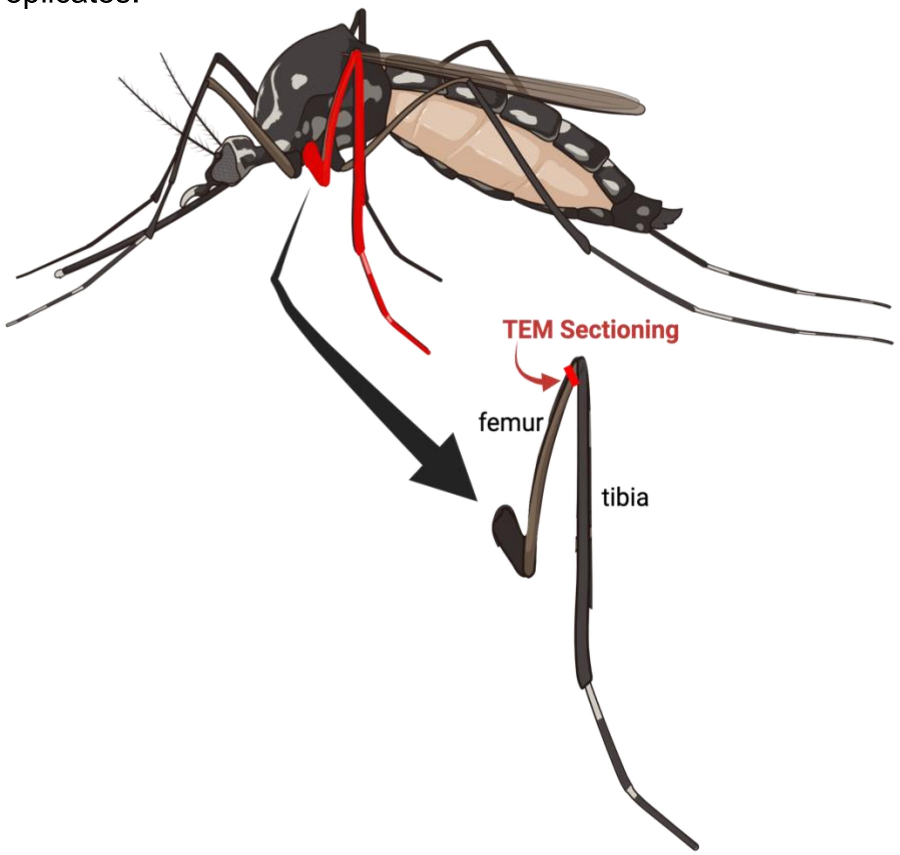

Supplementary Figure 2:
A: Schematic of TEM sectioning performed 200 nm into the midleg femur created using Biorender.

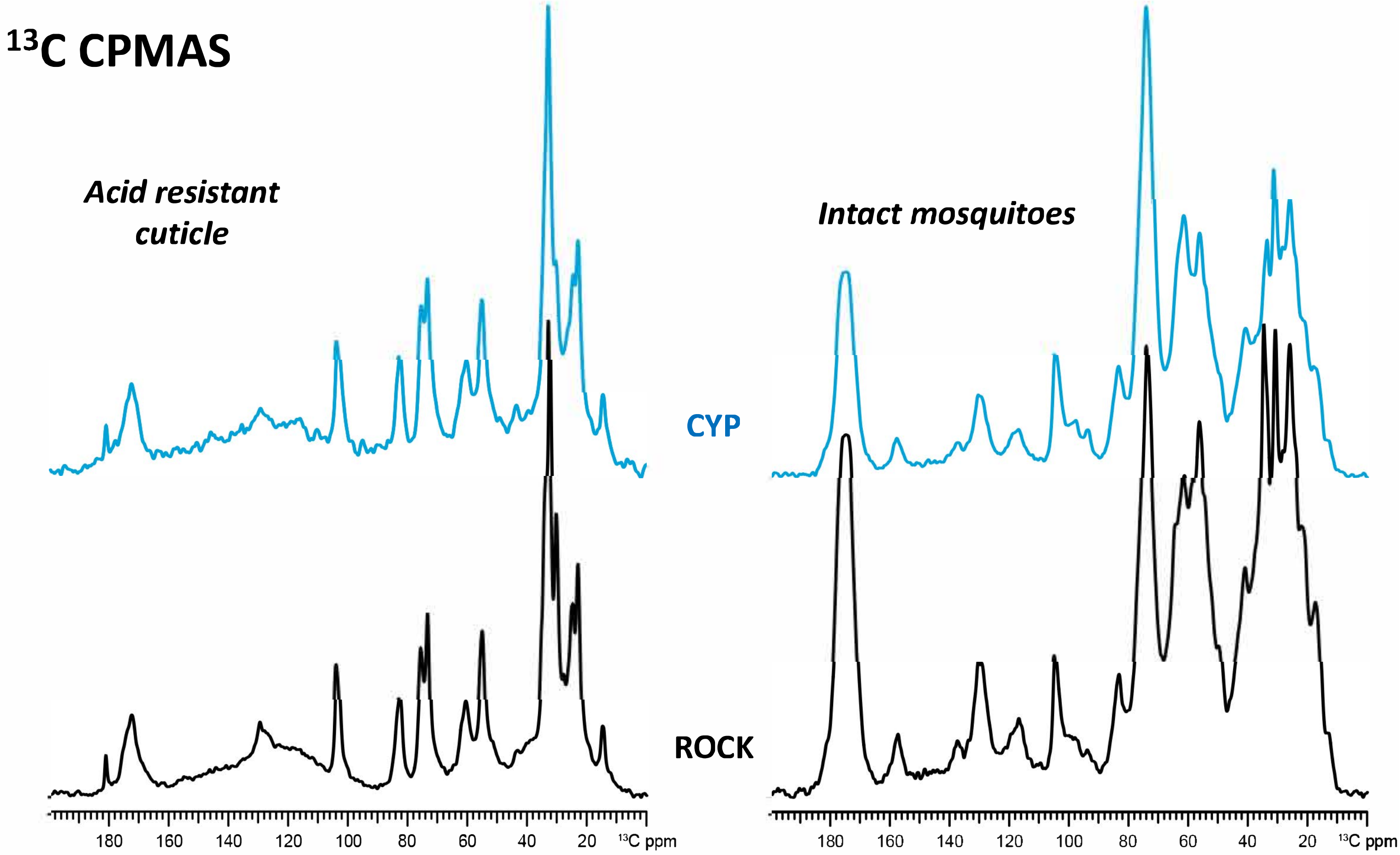

Supplementary Figure 3. ^{13}C cross-polarization (CPMAS) comparison of acid-resistant material (right) and whole female mosquitoes (left) of the CR (dark blue) and ROCK (grey) strains. The signals displayed are predominantly attributable to rigid moieties; the characteristic resonances of carbohydrate carbons (~55-105 ppm) and aromatic pigment carbons (~110-165 ppm) are clearly visible in the acid-resistant samples.

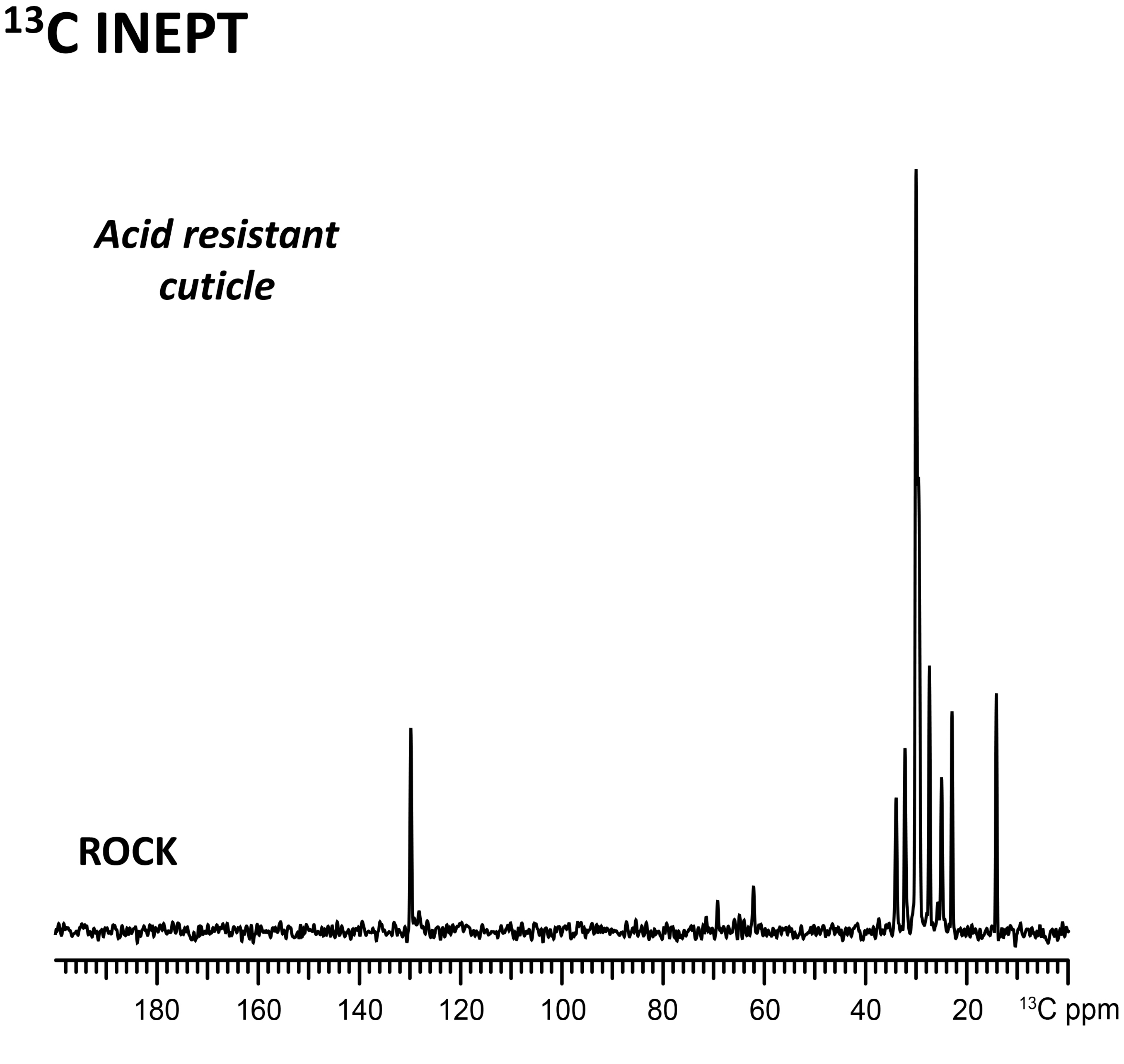

Supplementary Figure 4. ^{13}C INEPT (Insensitive nuclei enhancement by polarization transfer) spectrum of acid-resistant material from the ROCK strain. The signals displayed are exclusively attributable to triglycerides that undergo isotropic molecular motions on a timescale typical of rapidly tumbling molecules in solution.
